# Insights into the life strategy of the common marine diatom *Chaetoceros peruvianus* Brightwell

**DOI:** 10.1371/journal.pone.0203634

**Published:** 2018-09-12

**Authors:** Mirta Smodlaka Tanković, Ana Baričević, Ingrid Ivančić, Nataša Kužat, Nikola Medić, Emina Pustijanac, Tihana Novak, Blaženka Gašparović, Daniela Marić Pfannkuchen, Martin Pfannkuchen

**Affiliations:** 1 Center for Marine Research, Ruđer Bošković Institute, Rovinj, Croatia; 2 Department of Biology, University of Copenhagen, Helsingør, Denmark; 3 Department for Natural and Health Sciences, Juraj Dobrila University of Pula, Pula, Croatia; 4 Division for Marine and Environmental Research, Ruđer Bošković Institute, Zagreb, Croatia; Stazione Zoologica Anton Dohrn, ITALY

## Abstract

*Chaetoceros peruvianus* is a marine diatom species with circumglobal distribution. While frequently observed, it appears never to dominate the marine phytoplankton community hence it can be characterized as a rather opportunistic, generalistic species. Here we present ecological interpretations from a long-term data set on marine microphytoplankton in the northern Adriatic Sea, where the abundancies and relative contributions of *C*. *peruvianus* were observed along a set of steep ecological gradients. Limited supply of dissolved inorganic phosphate was identified as the driving ecological factor for this ecosystem. In parallel *C*. *peruvianus* was cultivated in monoclonal cultures and its morphological and physiological reaction to replete and phosphorus depleted medium was analysed. *C*. *peruvianus* reacted to phosphorus depletion by an increase in cell height and length as well as thickness and length of setae. This morphological reaction included an increase in cellular volume and calculated carbon content. Additionally, it represents the transition between two described morphological varieties, *C*. *peruvianus* and *C*. *peruvianus* var. *robusta*. *C*. *peruvianus* showed a significant induction of extracellular alkaline phosphatase activity if grown in phosphate depleted medium. Microscopical analysis demonstrated this activity to be located exclusively on the setae of the cells.

## Introduction

The diatom genus *Chaetoceros* Ehrenberg is one of the largest and most diverse diatom genera [1,2]. It contains more than 200 currently accepted species and more than 100 intraspecific names [2,3]. Overall, more than 300 taxa were described so far [1,3]. Characteristic morphological features of these centric diatoms are their setae, hollow spine-like structures, that protrude from the valve face or margin. Most species of the genus *Chaetoceros* form chains by either fusion of setae or direct attachments between valves. The diatom genus *Chaetoceros* is divided in two subgroups [2]: the Hyalochaete group has thinner setae that are void of chloroplasts while the Phaeoceros group is characterized by rather thick setae that bear chloroplasts and clearly a good portion of the cytoplasm. *Chaetoceros peruvianus* Brightwell is part of the Phaeoceros group and hence has rather thick and chloroplast bearing setae. This species generally does not form chains. Three varieties are described for the species [4]. *C*. *peruvianus* var. *currens* Paragallo is considered synonymous with *C*. *peruvianus* Brightwell [4]. *C*. *peruvianus* var. *gracilis* Schroeder is characterized by a rather elongated pervalvar axis and rather thin setae (2–3 μm) [4]. *C*. *peruvianus* var. *robusta* Cleve on the other hand shows similar measures for apical and pervalvar axes and rather thick setae of approximately 8 μm thickness [4]. Already Hustedt, but also recently Gomez considered *C*. *peruvianus* to be rather easily identifiable and records of its circumglobal distribution are probably reliable [4,5]. Such a circumglobal distribution of *C*. *peruvianus* observations is accumulated in the global biodiversity information facility GBIF [6,7] (S1 Fig). *C*. *peruvianus* is generally considered to be frequently observed but it never appears to grow to relatively high abundances like other bloom forming species that at times dominate their phytoplankton communities.

The northern Adriatic Sea is a highly structured and shallow ecosystem. Maximum depth is about 45 m. It is characterized by steep spatiotemporal ecological gradients [8,9]. These characteristics allow sampling strategies with reasonably good spatiotemporal coverage both across longitude and latitude as well as throughout the water column. This makes the northern Adriatic particularly suited for ecological observations of phytoplankton, as we can observe its behavior and succession while it travels through a multitude of ecological conditions. The phytoplankton of the northern Adriatic is largely dominated by diatoms [10] where nutrient availability is governed by the river Po, the largest freshwater input into the Mediterranean [11,12]. Our earlier results demonstrate that the phytoplankton succession and growth is mainly governed by light availability, temperature and the species capability to cope with phosphorus (P) limitation [13].

In situ measurements demonstrated that the expression of alkaline phosphatase activity as a tool to access the P from organic pools is a key response of the phytoplankton community to P limitation in the northern Adriatic [14,15]. Diatoms are known to express membrane bound, extracellular alkaline phosphatase when stressed by low concentrations of inorganic phosphate [16]. The enzymes dephosphorylate organic molecules and make the resulting phosphate available to the cells [17]. This mechanism is found to play a major role in marine phytoplankton adaptations to limited availability of dissolved inorganic phosphate [17,18]. A second prominent physiological response of diatoms to stress [19] by limitation of dissolved inorganic phosphate is a reduction of phospholipid content that is counterbalanced by an increase of non-phospholipids in the cells [20–22]. This mechanism is believed to increase growth capacity in phosphate limited conditions in the northern Adriatic [14,15,20]. Furthermore, experimental evidence in various microalgae demonstrates changes in expression levels of proteins involved in basic cellular metabolism as well as involved in the turnover of phosphorylated intermediary metabolites [23,24]. Cellular RNA content as well as ATP/Chlorophyll a ratio were also demonstrated to be reduced in response to phosphorous deprivation in microalgal cultures [25,26].

Furthering the knowledge on the metabolic reaction of diatoms to environmental stresses not only allows the prediction of their behavior in the environment, but also has implications on their application in biotechnology. Diatom metabolites are shown to have dramatic impacts on their environment [27] and are even demonstrated to control grazer reproduction [28]. Diatoms are prospected for biofuel production as well as bioactive substances with medical applications [29,30]. However the diatom metabolic activity as well a subsequent production of interesting metabolites is strongly growth phase depended and influenced or even triggered by nutrient deprivation [30]. We hence expect that the understanding of diatom reactions to environmental triggers not only helps us to understand their ecology and reaction to ecosystem changes but also helps us direct applied research approaches to the exploitation of diatom derived resources. We here characterize a common but non bloom forming diatom species.

As in other areas around the globe, *C*. *peruvianus*, is a common constituent of the marine phytoplankton community of the northern Adriatic Sea. The steep ecological gradients (in particular of nutrient availability) in the northern Adriatic and *C*. *peruvianus* in situ performance along those gradients gave us insight into possible factors governing its ecological behavior. Here, we present data from in situ measurements and observations of *C*. *peruvianus*. We furthermore investigate the cellular and physiological characteristics of *C*. *peruvianus* during a detailed time-course of P limitation by analyzing morphological features, alkaline phosphatase (AP) dynamics, P uptake dynamics and changes in the lipid composition. The current study provides a suite of cellular features, which serves as a basis for understanding ecophysiological behavior of natural P-limited not bloom forming taxa, particularly *C*. *peruvianus* populations.

## Materials and methods

### Sampling strategy

**Fig 1:**
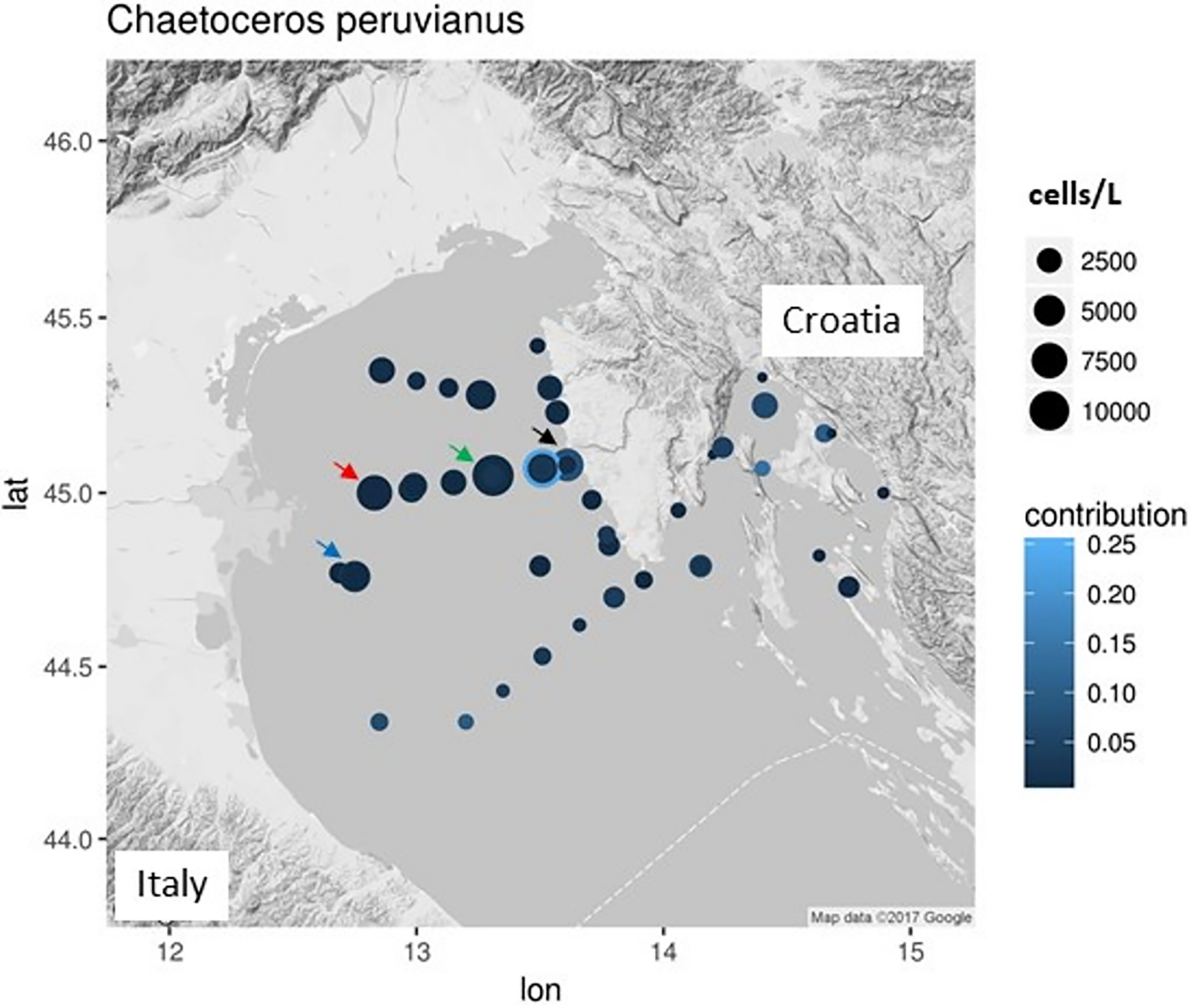
Spatial distribution of *C*. *peruvianus* in the northern Adriatic. The area of the circles marking the sampling positions represents the maximum abundance recorded for the position. The shade indicates the contribution relative to the total microphytoplankton abundances at sampling time. Arrows indicate the sampling stations analyzed for in situ alkaline phosphatase activity.

Samples were collected in the northern Adriatic Sea (NA) north of 44°N latitude (Fig 1). Sampling cruises were performed under the auspices and with permission of the Croatian Agency for Environment and Nature (CAEN). *C*. *peruvianus* spatial and temporal distribution was extracted from a long term monitoring data set containing 41 sampling positions with monthly to quarterly sampling frequency between the years 1972 and 2017 (Fig 1). Sampling followed the methodology described earlier [31]. In situ alkaline phosphatase activity was measured monthly at 4 stations (Fig 1) between November 2015 and October 2017 (see methods description below).

### Quantification of dissolved phosphate

PO_4_ concentration was determined with 20 μl of Vanadate-Molybdate reagent [32] added to 200 μl of cell cultures and measuring absorbance at 889 nm in the microplate reader (Infinite M200Pro, Tecan GmbH, Austria). Triplicates of each culture were measured. Standard curves of graded KH_2_PO_4_ solutions (concentrations ranging from 0.25 to 250 μM) were generated simultaneously with measurements. We calculated the cellular phosphate uptake as amount of dissolved phosphate removed from the culture medium during the growth period between two measurements divided by either the cell numbers at time of the measurement, or by the cell numbers at the time of the last measurement. Assuming that cell numbers grow between two measurements, the true cellular uptake rate for dissolved PO_4_ ought to be between both calculations.

### Lipid analysis

Total lipid and lipid class quantitation was performed by Iatroscan thin layer chromatography/flame ionization detection (TLC/FID) (Iatroscan MK-VI, Iatron, Japan). Triplicates of 80 mL culture, sampled at the end of growth (day 18), were filtered on precombusted 0.7 μm Whatman GF/F filters to determine the lipid composition of diatom *C*. *peruvianus*. The filters were stored at -80°C until lipid extraction. The particulate lipids were extracted by a modified one–phase solvent mixture of dichloromethane–methanol–water [33]. Five μg of hexadecanone (KET) was added to each sample before extraction. This internal standard was then extracted with the lipids in the sample, and the amount measured in the final concentrate provided an estimate of lipid recovery. Lipids were separated on silica-coated quartz thin-layer chromatography (TLC) rods (Chromarods SIII) (SES–Analysesysteme, Germany) and quantified by an external calibration with a standard lipid mixture. Each lipid class quantification was achieved using calibration curves obtained for representative standard by plotting peak area against the lipid amount spotted. Hydrogen flow rate was 160 mL min^-1^ and air flow rate was 2000 mL min^-1^. Each sample extract was analyzed in duplicate. For the analysis, 2 μl aliquots of 20 μl of the solution in dichloromethane were spotted by semiautomatic sample spotter. The standard deviation determined from duplicate runs accounted for 1–11% of the relative abundance of lipid classes.

The separation scheme of 18 lipid classes involves subsequent elution steps in solvent systems of increasing polarity. Quantified lipid classes include hydrocarbons (HC), lipid degradation indices (fatty acid methyl esters (ME), free fatty acids (FFA), alcohols (ALC), 1,3-diacylglycerols (1,3DG), 1,2-diacylglycerols (1,2DG) and monoacylglycerols (MG)), wax and steryl esters (WE/SE, further on discussed as SE (which are presumed to serve as inert storage forms of sterols) as in the phytoplankton monocultures WE are not supposed to be present as WE represent zooplankton storage lipids [34], phytoplankton energy reserves (triacylglycerols (TG)), membrane lipids including three phospholipids (phosphatidylglycerols (PG), phosphatidylethanolamines (PE) and phosphatidylcholines (PC)), glycolipids (sulfoquinovosyldiacylglycerols (SQDG), monogalactosyldiacylglycerols (MGDG) and digalactosyldiacylglycerols (DGDG)), sterols (ST) and pigments (PIG). For this work, we did not take into discussion lipid degradation indices. Total lipid concentrations were obtained by summing all lipid classes quantified by TLC-FID. A detailed description of the procedure is described in Gašparović et al. [21,35,36].

### In situ alkaline phosphatase activity

For quantification of alkaline phosphatase activity (APA) in situ, seawater was sampled with Niskin bottles and prefiltered through a 200 μm mesh to remove mesozooplankton. APA was measured in such filtered seawater using the fluorogenic substrate analogue methyllumbelliferyl-phosphate (MUF-P) at saturation concentration (50 μmol l^-1^), following the procedure described by Hoppe [37]. Aliquots of 2.5 mL, in triplicate, were used and incubation of the samples was performed in dark at in situ temperature and pH. Fluorescence was measured immediately after substrate addition and after ~1h of incubation using a Jenway fluorimeter with excitation 365 nm filter and emission 380–500 nm bandpass filter. APA was calculated as the difference between those measurements divided by the incubation time after calibration of the fluorimeter with methylumbelliferone, the product of MUF-P degradation. Species specific alkaline phosphatase activity in situ was analyzed as described earlier [15].

### Quantitative phytoplankton analysis

Phytoplankton samples (200 mL) were fixed with neutralized formaldehyde (2% final concentration). Phytoplankton cells were counted in 50 mL subsamples after 40 h of sedimentation time [38] using an Axiovert 200 microscope (Zeiss GmbH, Oberkochen, Germany) and following the Utermöhl [39] method. For distribution analyses of *C*. *peruvianus* we analyzed 9599 phytoplankton samples collected roughly monthly at 7 stations and roughly quarterly at 29 stations in the northern Adriatic between the years 1972 and 2017.

### Establishment of monoclonal cultures

Vertical net hauls were performed with a phytoplankton net (opening diameter 50 cm, length 2.50 m, mesh size 52 μm) from 15 m of depth to the surface. *C*. *peruvianus* cells were manually isolated with a micropipette from live net samples collected at various stations in the northern Adriatic Sea. Cells were grown into monoclonal batch cultures in 100 ml F/2 medium [40] and incubated at 18°C and 75 μmol photons m ^-2^ s ^-1^ on 12:12 h light/dark photoperiod.

### Genotyping

For molecular species identification, the 5’ end region of the ribulose bisphosphate carboxylase large subunit (*rbc*L) gene was used as a barcode. *C*. *peruvianus* cell cultures (25 mL) from the experiment were filtered on a 1,2 μm cellulose filter (Merck Millipore) and frozen at -80°C. Genomic DNA was isolated using the DNeasy Plant Mini Kit (Qiagen) according to manufacturer instructions. *rbc*L barcode was PCR amplified using the primer pair rbcL66+ (5’-TTAAGGAGAAATAAATGTCTCAATCTG-3’) and DtrbcL3R (5’-ACACCWGACATACGCATCCA-3’) [41,42]. Reaction mixture (25 μL) contained 200 μM of each dNTP, 0.3 μM of each primer, 4mM MgCl_2_, 1X DreamTaq Green buffer, 0.2 U of DreamTaq DNA polymerase (Thermo Scientific) and 0.5–1 ng of genomic DNA. The PCR reactions were performed in a C1000^TM^ Thermal Cycler (Bio-Rad Laboratories GmbH, Muenchen, Germany). PCR conditions were as follows: an initial denaturation step of 10 min at 95°C, 35 cycles of 30 s at 95°C, 30 s at 47°C and 1 min at 72°C, and final extension step of 7 min at 72°C. PCR amplified products were purified with MinElute PCR Purification Kit (Qiagen) according to manufacturer instructions. Purified PCR products were sequenced at Macrogen Europe (The Netherlands). The resulting sequences (from both ends) were aligned and further analyzed using Geneious 7.1.7. software [43]. BLAST was used for searches and comparisons of the NCBI GenBank database [44,45].

### Morphological analysis

Morphological features were observed in light microscopy (LM). All LM observations were carried out on field samples and exponentially growing cultures using a Zeiss Axiovert 200 microscope (Carl Zeiss, Oberkochen, Germany) equipped with Nomarski differential interference contrast (DIC), phase contrast, and bright-field optics. Light micrographs were taken using a Zeiss Axiocam digital camera. The terminology used to describe morphological features of *C*. *peruvianus* species follows Anonymous [46] and Ross et al. [47]. All the morphological measurements were made on LM micrographs in the software suite Axiovision 4.8 (ZEISS, Oberkochen, Germany). Biovolume of *C*. *peruvianus* cells was calculated using the following formula: V = π * (cellular width/2)^2^ * height + π*(width of setae/2)^2^ * total length of setae. Carbon content was calculated following the suggestions and results published by Menden-Deuer and Lesard [48] using the following formula: log pgC cell^-1^ = log a + b * log V (μm^3^) with log a = -0.933 and b = 0.881.

### In vitro cultures

200 mL of medium (F/2 or P-limit, see description below) were inoculated with 1 mL of a monoclonal culture (Center for Marine Research, Rovinj, Culture Collection, CIM 863). Batch cultures were followed through the course of the experiment. Each culture condition was prepared and followed in 3 independent replicates (triplicates). For each of the following parameters 3 independent measurements were performed. Results given are averages across triplicate measurements. For growth curve analysis, cell numbers of all three triplicate cultures were analyzed together to demonstrate the stability and significance of growth reactions to the medium conditions. Specific alkaline phosphatase activity as well as nutrient uptakes are given as values calculated across all measurements and all replicates for the respective culture condition.

Nutrient rich conditions were simulated by F/2 medium [40]. Stress by limitation of dissolved inorganic P was simulated in F/2 medium without sodium hydrogen phosphate (P-limit). Both media were prepared in NA seawater rested for 2 months in the dark, filtered twice through sterile 0.22 μm white plain filters (Merck Milipore Ltd.) and boiled in a microwave oven [49]. Media amendments were added through sterile filters. Cultures were incubated as batch cultures at 16°C at irradiance of 75 μmol photons m^-2^ s^-1^, each with a light dark cycle of 12:12 h in sterile 250 ml vented culture flasks (easy flasks, Nuclon, Denmark) from an initial concentration of 4.27 x 10^5^ cell L^-1^.

Cell concentrations were analyzed every second day of the experiment in Sedgewick-Rafter counting chambers (1 mL) on a Zeiss Axiovert 200 microscope. Morphological analysis was performed at the end of the exponential growth phase. Of each culture 5 subsamples of 20 μL were analyzed after DAPI staining on a Zeiss Axioimager fluorescence microscope using the Zeiss filterset 49 for epifluorescence as well as bright field phase contrast for transillumination [50]. Only cultures with no bacterial sized (0.2–3 μm) and DAPI positive particles were further analyzed.

### Growth curve

Growth curves were analyzed using non-linear fitting with the assumption of sigmoidal growth in batch cultures of the package Growthcurver in the software environment R [51,52].

### Alkaline phosphatase activity in vitro

APA in vitro was analyzed every second day in cultures as described earlier[15] but in this study modified for 96 well microplates. Substrate was added to *C*.*peruvianus* cultures. Final reaction volume was 250 μL. Product (methylumbelliferone) concentrations were detected by fluorescence intensity on a Tecan M200 Pro spectrofluorimeter (with excitation at 365 nm and emission at 460 nm) directly after the addition of the substrate and further after 10, 30 and 60 min of reaction time. Standard curves with concentrations ranging from 0.008 to 3 μM were generated for 4-methylumberlliferone (MUF; Sigma). Kinetic parameters were determined for the APA at 15 substrate concentrations between 0.5 μM and 400 μM. Results were analyzed using non-linear fitting of the package Dcr in the software environment R [53].

### Subcellular localization of alkaline phosphatase activity

Alkaline phosphatase activity was localized utilizing the ELF_97 Endogenous Phosphatase Detection Kit (E6601) (Thermo Fisher Scientific, Waltham USA) as described earlier [15,54,55]. Live cells from cultures where incubated with the alkaline phosphatase substrate. Microscopic analysis was performed using a Zeiss Axiovert Epifluorescence microscope. Chloroplasts were detected by their autofluorescence (Filterset 14 Zeiss), and insoluble fluorescencent product of alkaline phosphatase activity was localized using a specially adapted filter set (exitation: 340/26, beamsplitter 400 longpass, emission 525/50).

### Statistical analyses

Growth curves were analyzed using the R packages Growthcurver and Ggplot2 [52,56]. Multiple analysis of variance was performed using the function MANOVA and t-tests (Welch’s Two Sample t-test) were performed using the function t-test of the R base package [51].

## Results

### Species identification

*C*. *peruvianus* was morphologically identified using light microscopy and following the morphological description by Hustedt [4]. Using the *rbc*L gene as a DNA barcode we confirmed the isolated culture as *C*. *peruvianus*. Nucleotide BLAST similarity search found only one *rbc*L sequence identified as *C*. *peruvianus* in the GenBank database [57]. The *C*.*peruvianus* sequence reported here shows 98% pairwise identity (on 99% query cover) with that sequence. The here reported *C*. *peruvianus rbc*L nucleotide sequence was deposited in GenBank under Accession number: MH393890.

### Spatio-temporal distribution of *C*. *peruvianus* in the northern Adriatic

*C*. *peruvianus* was found at 38 sampling stations across the northern Adriatic (Fig 1). Abundances ranged between 39 and 11100 cells L^-1^, with a 1^st^ quartile at 370 cells L^-1^ a median at 740 cells L^-1^, a mean at 1071 cells L^-1^ and a 3^rd^ quartile at 1480 cells L^-1^. Highest abundances (up to about 11100 cells L^-1^) were recorded in the north-western part of the northern Adriatic, with highest records along a transect across the northern Adriatic at roughly latitude 45 degrees north.

**Fig 2:**
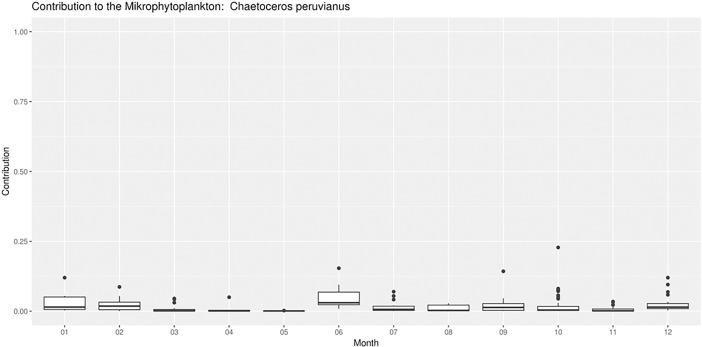
Box and whisker plot of the contributions of *C*. *peruvianus* throughout the year.

Contributions of *C*. *peruvianus* to the total microphytoplankton abundance ranged between 0.0001 and 0.2280 (October 2000), with a 1^st^ quartile at 0.002, a median at 0.006, a mean of 0.0208 and a 3^rd^ quartile at 0.0279. Fig 2 shows the contributions of *C*. *peruvianus* throughout the year. Highest contributions were found in July and in January, with some rare exceptional numbers in October.

## Growth dynamics

Cultures in full medium (F/2) as well as in F/2 medium without added PO_4_ (P-limit) were started at cell concentrations of 42x10^4^ cells L^-1^. In both media types, the cultures reached the beginning of a stationary phase after 18 days, when they reached average concentrations of 19.7x10^6^ cells L^-1^ (stdev = 47x10^4^) in F/2 medium and 15.1x10^6^ cells L^-1^ (stdev = 13x10^5^) in P-limit medium.

**Fig 3:**
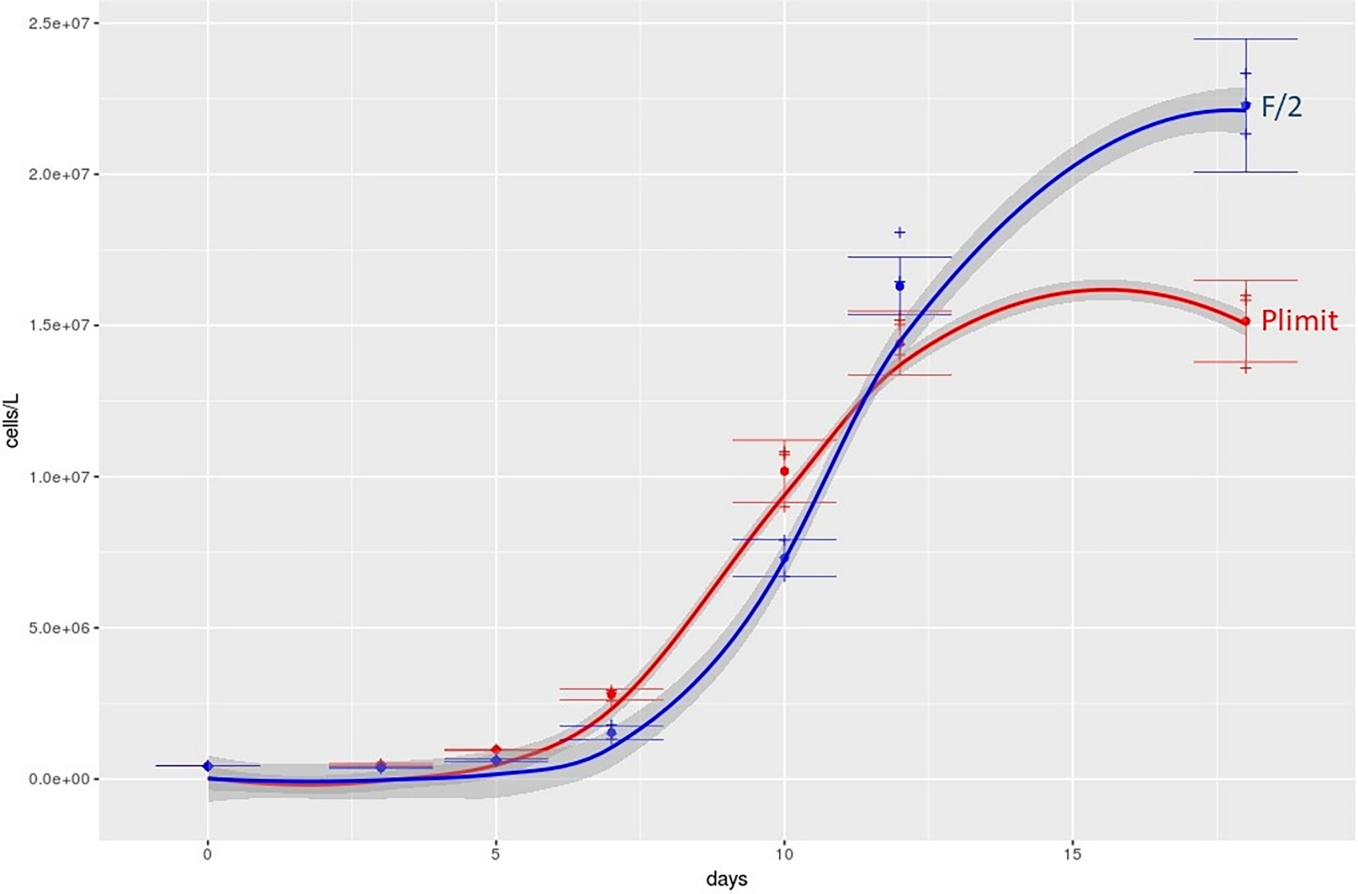
Growth curves of *C*. *peruvianus* in different phosphate conditions. Growth curve in F/2 medium is indicated in blue and growth curve in F/2 medium without added dissolved phosphate (P-limit) in red. Averaged cell concentrations throughout 18 days of culture duration and fitted models for growth in batch cultures are presented. Crosses are measured cell concentrations, dots give the averages for the given medium. Lines show the resulting model values and the grey area around the lines represent the 95% confidence interval for the respective model.

Fig 3 shows the growth curves, averaged cell concentrations throughout 18 days of culture duration as well as fitted models for growth in batch cultures. The cultures in F/2 reached the inflection point of the growth curve (shortest generation time) on average after 10.90 days, while the cultures in P-limit reached that inflection point on average after 9.15 days. Shortest generation time for cultures in F/2 was on average 0.81 days, while shortest generation time for cultures in P-limit was 0.851 days. Multiple analysis of variance on inflection points and minimum generation times revealed a significant difference (Pr < 0.0014) between two culture conditions.

### Morphological reactions to stress by low dissolved inorganic phosphate concentrations

**Fig 4:**
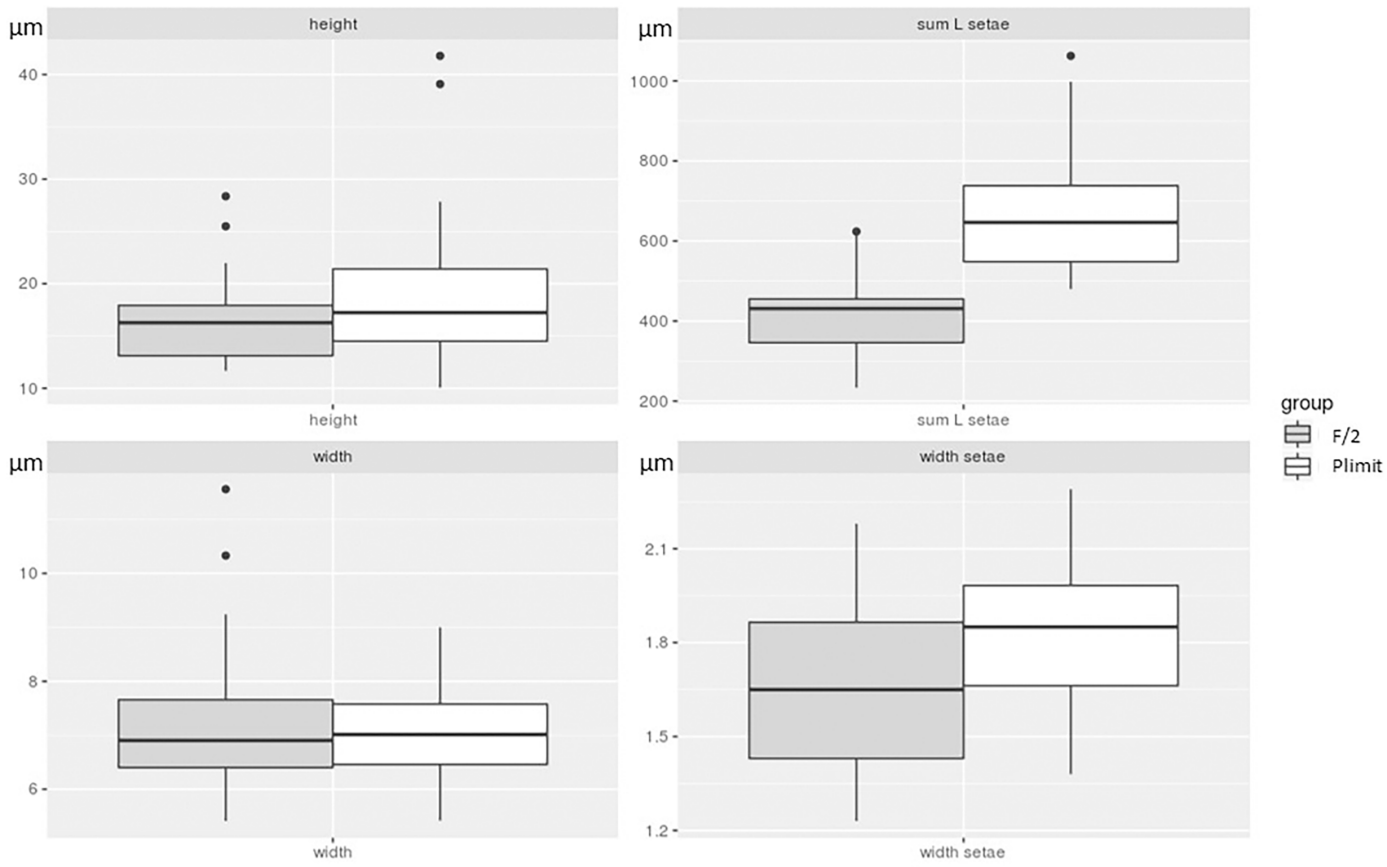
Morphology measurements. Box and whisker plots for the cell height (pervalvar axis), cell width (apical axis), sum lengths of all setae per cell and width of setae for *C*. *peruvianus* grown in F/2 (grey) and P-limit (white) medium. For both media 150 cells were measured.

**Fig 5:**
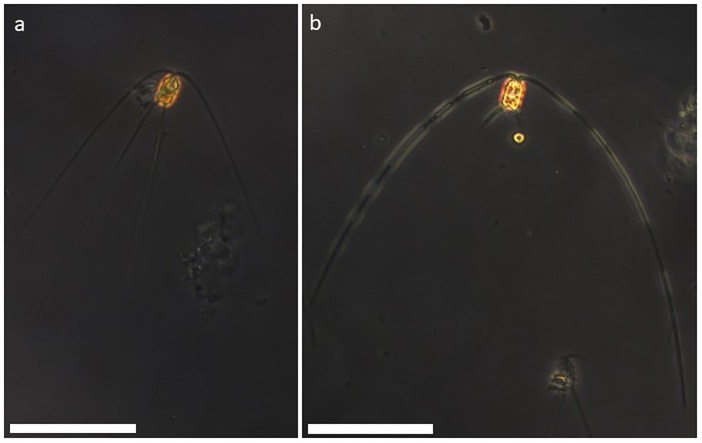
Light microscopy of *C*. *peruvianus*. Cells of *C*. *peruvianus* grown in F/2 medium (a) and in P-limit medium (b). Note the thickened and elongated setae of the cell grown in P-limit medium (b). Scale bar represents 20 μm.

*C*. *peruvianus* cells grown in P-limit medium showed marked morphological differences if compared to cells grown in F/2 medium. Fig 4 shows the 4 cell measures analysed for the culture conditions F/2 and P-limit. A Welch two sample t-test revealed significant differences for cell height (pervalvar axis) (p-value = 3.893x10^-3^), width of setae (p-value = 0.01027) as well as for the sum of the length of all 4 setae per cell (p-value = 4.731x10^-9^). Average cell height was 16.47 μm and 19.28 μm for cells grown in F/2 or P-limit media respectively. Average overall length of setae per cell was 418.07 μm and 672.06 μm for cells grown in F/2 or P-limit media respectively. Average width of setae was 1.65 μm and 1.83 μm for cells grown in F/2 or P-limit media respectively. Fig 5 shows *C*. *peruvianus* cells grown in F/2 (a) and Plimit (b) medium and demonstrates the thickened and elongated setae of cells grown in P-limit medium. Calculated cellular biovolume (from average values) was 1580.17 μm^3^ and 2503.70 μm^3^ for cells grown in F/2 and P-limit medium, respectively.

### The dynamics of dissolved inorganic phosphate uptake

**Fig 6:**
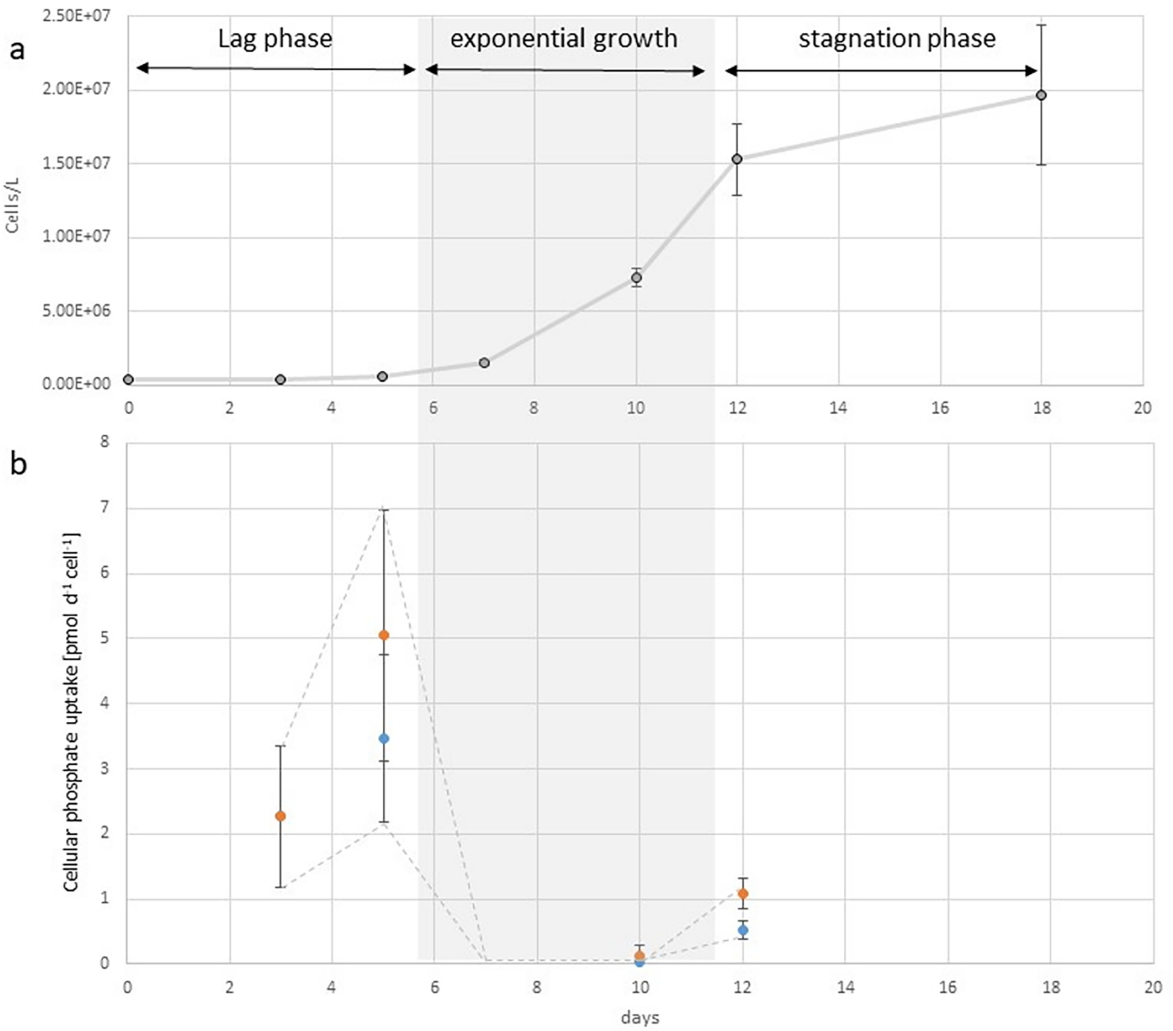
Dynamics of phosphate uptake rates throughout batch cultures of *C*. *peruvianus* in F/2 medium. (a) Average cell concentrations (and standard deviations) plotted over the duration of the batch culture.in days. The phases of the growth curve are indicated. (b) Cellular phosphate uptake rates calculated as phosphate uptake divided by cell numbers at the days of phosphate quantification (blue, or lower values) and divided by the cell numbers at the day of the measurement before (orange, or higher values). The dashed line indicates lower and upper borders between the two calculation methods. The true uptake rate must be between the two borders. Note the steep drop of phosphate uptake rates during exponential growth.

Calculated cellular PO_4_ uptake rates in F/2 medium were between 0.01 pmol cell^-1^ d^-1^ and 5.04 pmol cell^-1^ d^-1^. Dissolved phosphate concentrations were below detection limit (0.02 μM) in P-limit cultures throughout the experiments, so no phosphate uptake rates were calculated for those cultures. Fig 6 shows the dynamics of phosphate uptake rates throughout the experiment duration of batch cultures in F/2 medium. We detected high and increasing phosphate uptake rates during the lag phase of the batch culture growth, followed by a steep decrease and very low uptake rates during the exponential growth phase and a slight increase of phosphate uptake rates at the beginning of the batch culture stationary phase.

### Alkaline phosphatase activity

**Fig 7:**
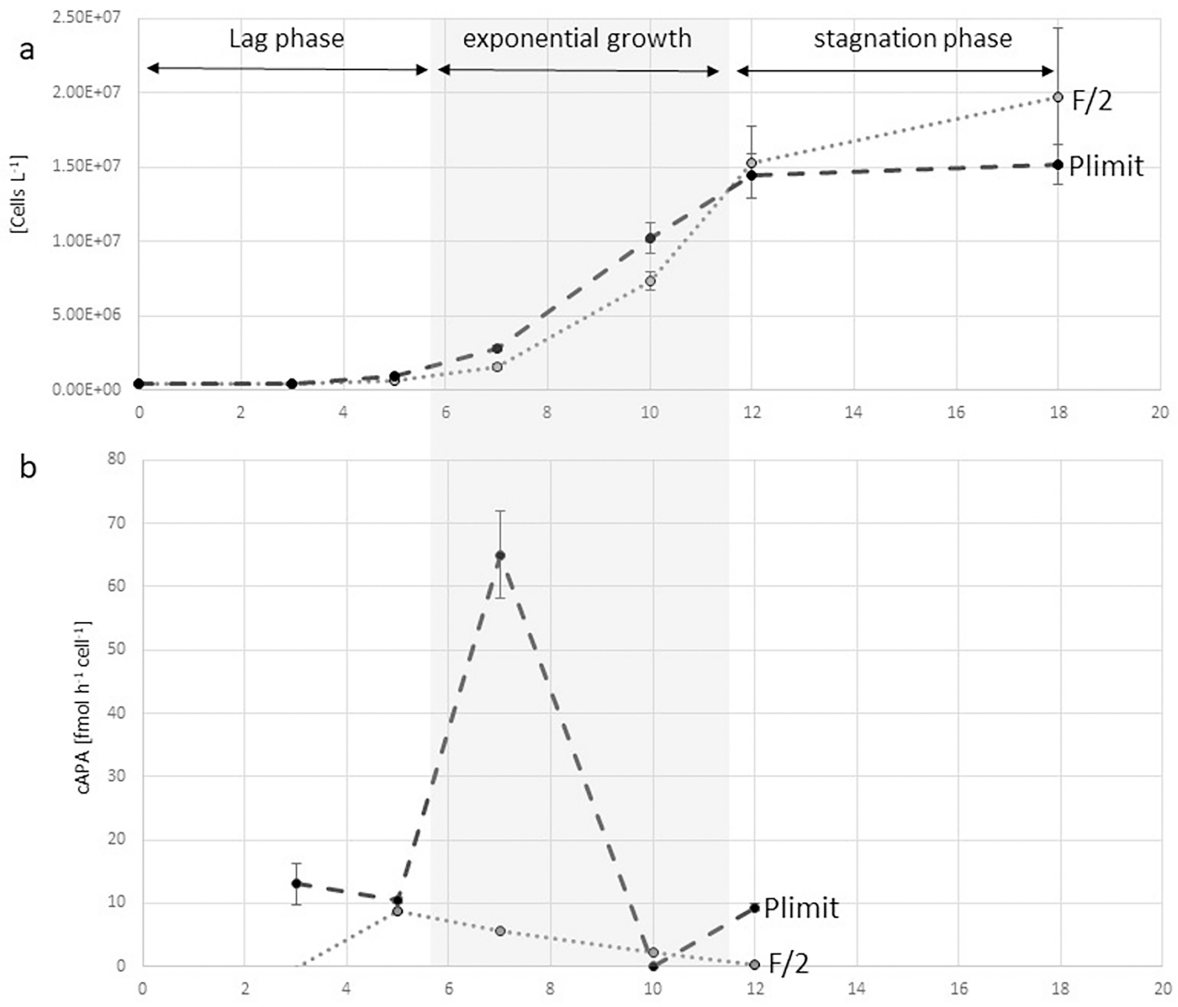
Dynamics of cellular alkaline phosphatase activity (APA) during batch cultures of *C*. *peruvianus* cells in F/2 medium and in P-limit medium. (a) Growth curves (average cell numbers with standard deviations as error bars) in F/2 medium (dotted line) and in P-limit medium (dashed line). (b) Cellular APA in F/2 medium (dotted line) and in P-limit medium (dashed line). Note the sharp in increase at the end of the lag phase and the steep reduction of cellular APA during the exponential growth phase.

For in vitro experiments, we calculated cellular APA as measured APA activity divided by the number of cells in the sample. We detected a maximal APA of 65 fmol h^-1^ cell^-1^ at the beginning of the exponential growth phase in P-limit. Fig 7 shows the dynamics of APA across the growth curves of cultures in F/2 and P-limit medium. Low cellular APA of around 8 fmol h^-1^ cell^-1^ was measured in F/2 medium during the lag phase and the beginning of the exponential growth phase. This activity dramatically fell during the exponential growth phase and stayed very low during the beginning of the stationary phase. In P-limit medium we observed a dramatic increase in APA up to 65 fmol h^-1^ cell^-1^ at the beginning of the exponential growth phase. During the exponential growth phase APA per cell was reduced dramatically and then increased during the beginning of the stationary phase again to about 10 fmol h^-1^ cell^-1^. The analysis of the AP enzyme kinetics showed a half saturation constant (Km) value of 64.59 μM (standard error = 17.97, Pr = 0.000834) which indicates a low affinity enzyme.

**Fig 8:**
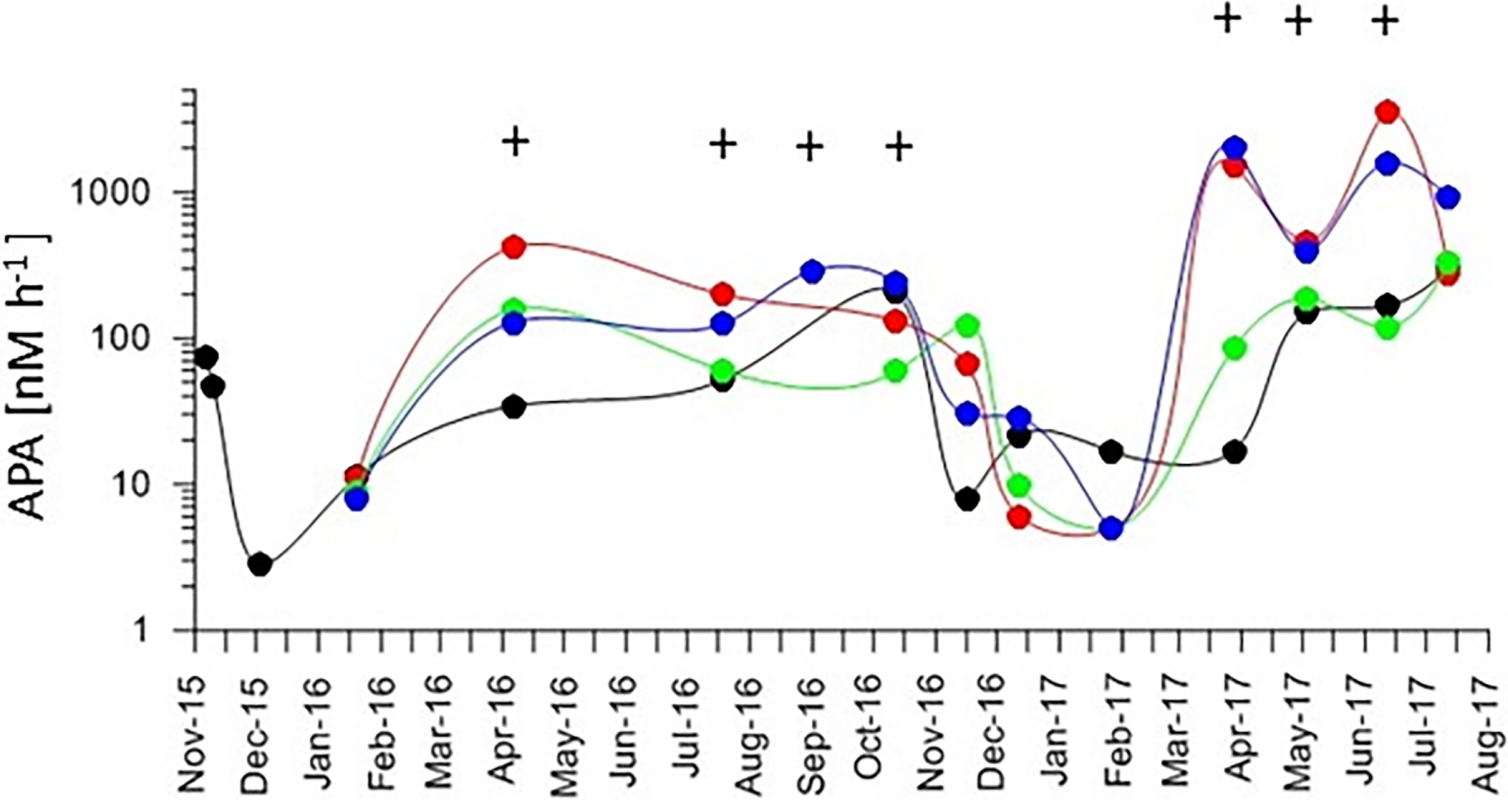
Alkaline phosphatase activity in water samples between November 2015 and August 2017 at different longitudes across the northern Adriatic. Blue (12.75), red (12.83), green (13.31), black (13.61) (see positions indicated by correspondingly colored arrows in Fig 1). Crosses mark sampling dates when *C*. *peruvianus* cells were found in in situ samples to show extracellular alkaline phosphatase activity. Their activity pattern followed the general trend of alkaline phosphatase activity in water samples throughout the year.

In situ analysis of extracellular APA by *C*. *peruvianus* was performed in parallel to determination of total APA in seawater samples (including phytoplankton organisms at natural concentrations). On 4 stations across the northern Adriatic (see arrows in Fig 1) we found a stable pattern of APA in seawater that rose in spring during the onset of stratification and fell in late autumn during the onset of water column mixing (Fig 8). Black crosses indicate sampling dates, when microscopic analysis showed *C*. *peruvianus* cells with extracellular alkaline phosphatase activity.

### Localization of alkaline phosphatase activity

**Fig 9:**
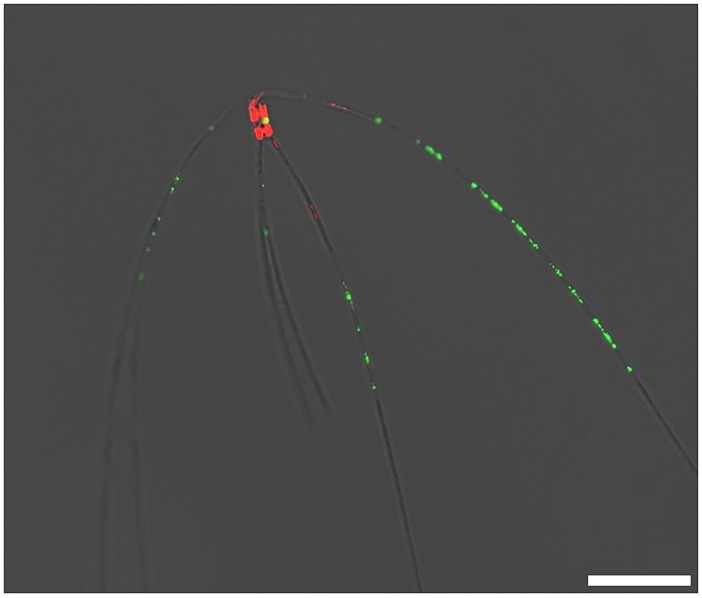
*C*. *peruvianus* cell from a culture in P-limit medium after incubation with alkaline phosphatase substrate from the ELF 97 Phosphatase Detection Kit. Chloroplast autofluorescence is shown in red. The fluorescent signal from dephosphorylated alkaline phosphatase substrate is shown in green. It accumulates at the location of alkaline phosphatase activity, along the setae of the cell. Scale bar represents 20 μm.

After incubation of *C*. *peruvianus* cells with substrate for AP from the ELF 97 Endogenous Phosphatase Detection Kit, the fluorescent product accumulated on the setae of *C*. *peruvianus* cells. Fig 9 shows an exemplary cell grown in P-limit medium. Notably all of the green signal for APA is localized on the setae.

### Cellular lipid composition

Table 1 presents the *C*. *peruvianus* cellular content of the lipid classes identified and their percentage contribution to total lipids for the P-replete (F/2) and P-limited (P-limit) culturing conditions. No significant difference in neither lipid and lipid class content nor in the percentage contribution to total lipids between the cells grown in F/2 and in P-limit medium could be found (P>0.05 for all distinguished lipid classes, see Table 1).

**Table 1:** Cellular content of the lipid classes (pg cell^-^1) identified and their percentage contribution to total lipids (%) for the P-replete (F/2) and P-limited (P-limit) culturing conditions. Standard deviations (Stdev) for culture triplicates are indicated. Phosphoglycerols (PG), phosphatidyl ethano amin (PE), phosphatidylcholine (PC), monogalactosyldiglyceride (MGDG), di galactosyl diglyceride (DGDG), sulfoquinovysil diglyceride (SQDG), sterols (ST), pigments (PG), hydrocarbons (HC), steryl esters (SE), triglycerides (TG). The last row (P t-test) shows P values for a Welch two sided t-test (F/2 against P-limit).

	Lipid_T_	PG	PE	PC	MGDG	DGDG	SQDG	ST	PIG	HC	SE	TG
	pg cell^-1^											
**F/2**												
	55.63	9.27	9.07	0.47	8.12	1.49	6.38	3.03	1.75	3.40	0.71	11.95
	45.85	14.87	7.52	0.41	2.67	1.07	8.95	1.58	0.76	2.66	0.29	5.07
	63.69	15.65	7.95	0.71	6.17	1.23	5.94	3.31	1.50	1.64	0.68	18.91
Av.	55.06	13.26	8.18	0.53	5.65	1.26	7.09	2.64	1.34	2.57	0.56	11.98
Stdev	8.93	3.48	0.80	0.16	2.76	0.21	1.63	0.93	0.52	0.88	0.23	6.92
%		24.09	14.86	0.96	10.27	2.29	12.87	4.79	2.43	4.67	1.01	21.75
Stdev(%)		3.48	0.80	0.16	2.76	0.21	1.63	0.93	0.52	0.88	0.23	6.92
**Plimit**												
	83.88	13.25	11.71	1.03	9.22	2.93	7.94	3.57	4.37	1.96	0.61	27.30
	52.49	13.76	13.36	0.42	4.22	1.18	5.87	1.93	1.98	0.68	0.83	8.26
	73.19	16.76	23.68	1.31	5.45	0.67	8.37	0.86	2.10	2.58	0.74	10.67
Av.	69.85	14.59	16.25	0.92	6.29	1.60	7.39	2.12	2.82	1.74	0.73	15.41
Stdev	15.96	1.90	6.48	0.45	2.60	1.19	1.34	1.36	1.34	0.97	0.11	10.37
%		20.89	23.27	1.32	9.01	2.29	10.58	3.03	4.03	2.49	1.04	22.06
Stdev(%)		1.90	6.48	0.45	2.60	1.19	1.34	1.36	1.34	0.97	0.11	10.37
P t-test	0.08	0.23	0.09	0.09	0.23	0.31	0.44	0.32	0.07	0.23	0.24	0.33

## Discussion

### Spatio-temporal distribution of *C*. *peruvianus* in the northern Adriatic

The northern Adriatic is the shallow and most northern part of the Mediterranean. Under the influence of the Po river steep spatio-temporal gradients of nutrient availability are formed, both longitudinally as well as latitudinally across the northern Adriatic [8,58]. Availability of PO_4_ decreases eastwards and southwards in the basin and exerts a strong influence on the phytoplankton community structures observed [10,14,15,59]. Hot summer temperatures heat the water column up to 30°C (with a thermocline formation at around 15 m depth and significant lower temperatures below the thermocline), while strong and cold winds during winter season cool the water column down below 10°C [8,9,60,61]. A general cyclonic current system in the northern Adriatic not only stabilizes the ecological aforementioned gradients, but also transport the northern Adriatic phytoplankton along those gradients, including advection of plankton from the middle Adriatic in the eastern part of the basin and an outflow of northern Adriatic plankton southwards along the western coast of the Adriatic. Consequently, the northern Adriatic phytoplankton can be observed under a variety of different ecological conditions [8,9,11,14]. Our results show highest abundances of *C*. *peruvianus* on a transect across the northern Adriatic at latitude 45° north (Fig 1). This coincides with the area where nutrients, transported with the river Po into the northern Adriatic, are spread across the basin which resulted in maxima of phytoplankton abundances. Phytoplankton blooms develop at the western part of the basin, near the Po river mouth, where dissolved nutrients are taken up very fast while nutrient depleted water is transported with the current towards the eastern Adriatic coast during surface temperature induced water column stratified period. However, maximal contributions of *C*. *peruvianus* to the phytoplankton community were observed at the eastern part of the study area, where generally nutrient concentrations and phytoplankton abundances are lower. Not once *C*. *peruvianus* was observed to dominate the phytoplankton community, which demonstrates, that there are always other phytoplankton species that outcompete *C*. *peruvianus* under the given conditions in the northern Adriatic. Fig 2 shows highest contributions of *C*. *peruvianus* in January, during the winter clear water period, when the water column is mixed entirely and the autumn phytoplankton bloom ended already. A second peak contribution can be observed in June after the spring phytoplankton bloom. The aforementioned spatial and temporal distribution of abundance and contribution values suggest, that *C*. *peruvianus* is opportunistically present in most conditions observed in the northern Adriatic. However, the species appears to be most successful in post phytoplankton bloom periods and at lower temperatures, when general phytoplankton production is relatively low. In those conditions organic phosphate is relatively abundant, while inorganic dissolved phosphate is a limiting factor [8,9,11,14,15].

### Growth dynamics

To understand how *C*. *peruvianus* grows in nutrient replete conditions and how the species reacts to PO_4_ limitation, it was grown in F/2 and P-limit medium. Our analyses showed a significantly, but only slightly altered growth curve when inorganic phosphate was kept below 0.2 μM in the medium (Fig 3). In P-limit medium, the lag phase was slightly shortened, maximum growth rates as well as maximum abundances and stagnation phase were reached earlier and maximal cell concentrations were lower if compared to F/2 medium. Overall, *C*. *peruvianus* appears to grow better and faster and to higher concentrations when nutrients are abundantly available. However, those differences between the two media are marginal if compared to reactions of other diatom species [26,62,63]. It would hence appear, that phosphate availability is no particularly strong trigger for alterations in growth dynamics. This nicely fits the in situ observations, where *C*. *peruvianus* abundances and contributions are always moderate, and neither nutrient inputs nor limitations correlate with any dramatic changes in observed abundances. The shortest observed generation time (maximum growth rate) was 0.81 days, which is rather common for diatoms [64–68]. The observed maximal growth rates are in the range of a calculated possible maximal growth rate that is derived from possible carbon accumulation through RuBisCO activity [65,69]. It hence appears that *C*. *peruvianus* in principal can grow as fast as other species, but either increased growth rate is not induced in situ or competing species have faster nutrient uptake mechanisms which keeps *C*. *peruvianus* from outcompeting other species in situ.

### Morphological reactions to stress by low dissolved inorganic phosphate concentrations

*C*. *peruvianus* cell grown in P-limit medium showed marked morphological differences if compared to cells grown in F/2 medium (Figs 4 and 5). The morphological alterations in P-limit medium include a significantly elongated pervalvar axis, as well as significantly increased width and length of setae if compared to the morphological characteristics of cells grown in F/2 medium. This results in an overall increase in cellular volume. The morphometrics of cells grown in F/2 medium fall within the ranges from the original species description elaborated on by Hustedt [4]. However, the morphometrics of cells grown in P-limit medium resemble the morphological description of *C*. *peruvianus* var. *robusta* Cleve. Our experimental results hence demonstrate that one clone of *C*. *peruvianus* can change its morphology as a reaction to nutrient availability so much that it resembles *C*. *peruvianus* var. *robusta* Cleve. This is a good indication that in fact *C*. *peruvianus* var. *robusta* Cleve should be considered synonymous with *Chaetoceros peruvianus* Brightwell. Furthermore, these results demonstrate that morphometric analysis of *C*. *peruvianus* in situ samples might deliver a good indicator for the ecological conditions the cells grew in. Morphometrics resembling those of *C*. *peruvianus* var. *robusta* Cleve might indicate limited availability of PO_4_. It however remains to be investigated if those morphological peculiarities can be evoked by other limitations as well.

### Localization of alkaline phosphatase activity

The ELF 97 Endogenous Phosphatase Detection Kit contains a non fluorescent, water soluble substrate for alkaline phosphatase. After dephosphorylation a fluorescent and non soluble product is formed, which accumulates at the site of phosphatase activity [70]. In our experiments, the fluorescent product accumulated exclusively on the setae of the *C*. *peruvianus*. This observation suggests that the localization of membrane bound extracellular alkaline phosphatase as well as the respective activity is limited to the setae of the species (Fig 8). The abovementioned morphological reactions observed in P-limit medium show an increase of volume and surface in particular the setae, where APA is located. It hence appears that under limited availability of PO_4_, *C*. *peruvianus* increases the surface area where APA is located to increase its capability of using organic phosphate sources. There is much speculation about the function of setae so far. While increasing the cells dimensions they might be obstructive to predators or might alter the cells hydrodynamic characteristics and hence decrease sinking rates [71]. However, our results show that one function of the setae is to provide surface area for membrane bound extracellular enzyme activity like APA. This surface area can even be increased when the environmental conditions demand increased APA.

### The dynamics of dissolved inorganic phosphate uptake

Phytoplankton, while drifting in the northern Adriatic is exposed to quickly changing nutrient regimes. Localized nutrient inputs like rivers or heavily populated coasts as well as general nutrient increases during either localized upwelling or mixing of the water column produce spatio-temporal limited peaks of nutrient availability [72,73]. The capability to quickly internalize or make use of such nutrient peaks is an important factor for successful competition in the coastal phytoplankton [14,74]. We observed a maximal uptake rate of 5.04 pmol cell^-1^ d^-1^ which is comparable to uptake rates reported for other phytoplankton species [75]. The dynamics of the cellular phosphate uptake rates throughout the growth curve is particularly interesting. Highest uptake rates were observed at the end of the lag phase of the cultures. With the beginning of the exponential growth phase cellular uptake rates dropped dramatically to 0.01 pmol cell^-1^ d^-1^ and only rose again at the end of exponential growth phase and the beginning of the stationary phase (Fig 6). It hence appears that in stationary phase, during cell cycle arrest (G0) phosphate, uptake rates are highest, while during intense cell division and shortened G1, S and G2 phases in the cell cycle, when cell contents are duplicated, as well as during cell division phosphate cellular uptake rates are very much reduced. In situ this would mean that *C*. *peruvianus* when faced with sudden availability of PO_4_ during times of slow or no growth would benefit from a high cellular phosphate uptake rate (for the species). However, during a potential bloom formation or fast growth period cellular phosphate uptake rates would drop and allow for increased growth rates also when the availability of PO_4_ quickly decreases e.g. when other species with fast bloom formation used up all available PO_4_. This would fit the in situ observation that *C*. *peruvianus* generally achieves higher contributions in the eastern oligotrophic part of the northern Adriatic, where the phytoplankton of the northern Adriatic drifts after fast blooming species used up inorganic dissolved phosphate and already start to release organic material including organic phosphate as a result of either cell death or grazing [11].

### Qualitative and quantitative analysis of cellular lipid content

Neither total lipid nor any lipid classes cellular content appeared to show significant differences between *C*. *peruvianus* grown in P-replete (F/2) and P-limit culturing conditions. This would suggest that *C*. *peruvianus* growing in the P limited conditions does not employ a strategy to support intracellular P requirements (for e.g. DNA, RNA, ATP) by exchanging membrane phospholipids for sulfolipids [22]. For PE we found a higher contribution in cells grown under P limited conditions. PE are usually considered lipids of bacterial origin [76]. However, PEs are also found in some diatoms [77]. Our results hence support the notion that also diatoms have to be considered a source for PE.

Unlike often reported for other species, where cellular TG content increases when diatom cultures become P depleted [78,79]. *C*. *peruvianus* does not adjust carbon metabolism to synthesize extra TG in phosphorus scarcity. Very likely *C*. *peruvianus* is channeling carbon from primary production to a cell volume increase rather than creating reserves. This inability of *C*. *peruvianus* to adjust lipid metabolism during growth in P-limit conditions further explains the in situ observations, where the species never dominates (outcompetes) the phytoplankton community. This might be due to the inadequate physiological response, compared to more competitive species that use alternative lipid compositions as a strategy to compete in changing environmental conditions.

### Alkaline phosphatase activity

Cellular APA was maximal with 65 fmol h^-1^ cell^-1^ at the beginning of the exponential growth phase. Fig 7 shows the dynamics of APA across the growth curves of cultures in F/2 and Plimit medium. In P limited conditions, cellular APA was induced early during the lag phase of the cultures and quickly increased to its maximum during the beginning of the exponential growth phase. We did measure low APA of around 8 fmol h^-1^ cell^-1^ in F/2 medium during the lag phase and at the beginning of the exponential growth phase. This activity dramatically fell during the exponential growth phase and stayed very low during the beginning of the stagnation phase. In P-limit medium we observed a strong increase in APA up to 65 fmol h^-1^ cell^-1^. Maximum APA was reached in P-limit cultures at the beginning of the exponential growth phase as well. During the exponential growth phase APA per cell was reduced dramatically and then increased during the beginning of the stagnation phase again to about 10 fmol h^-1^ cell^-1^. Like for the cellular phosphate uptake rate it appears, that during cell cycle arrest or G0 phase the cells can accumulate maximal amounts of alkaline phosphatase on their cell surfaces, while this enzyme appears to be not produced or exported during intensified and shortened G1, S and G2 phases, which results in a reduction of alkaline phosphatase per cell during cell division. For in situ conditions this might translate into intensified cellular APA during the initialization of fast growth periods (or blooms) of *C*. *peruvianus*, while during the phases of fast growth or bloom formation cellular APA is strongly decreased. The characterization of the alkaline phosphatase enzyme kinetics showed a very high K_m_ value of 64.59 μM. This is a comparatively high K_m_ if considering that most species in the northern Adriatic show a K_m_ value of around 1 μM or below. Such a high K_m_ indicates a low specificity for substrate and probably indicates moderate or low performance in the competition for organic phosphate when substrate concentrations are low, which might be another reason why *C*. *peruvianus* does not reach higher contributions to the microphytoplankton community, where dominating species probably induce high specificity AP.

In situ data showed that *C*. *peruvianus* followed the general trend of APA of marine plankton in the northern Adriatic (Fig 8). APA closely correlates with the stratification pattern of the water column in the northern Adriatic [14,15,80,81]. During unstratified conditions PO_4_ is available and primary production is low, while during stratified conditions primary production is high and concentrations of dissolved PO_4_ are minimal, while organic phosphate becomes an important source for phytoplankton.

## Conclusions

*C*. *peruvianus* is a common marine diatom with global distribution. Wide range of ecological conditions allow the persistence and survival of *C*. *peruvianus*. In the northern Adriatic *C*. *peruvianus* achieves higher contributions to the phytoplankton and hence competes more successfully under conditions when inorganic phosphate resources are depleted and organic phosphate concentrations increased. Relatively high Km of its alkaline phosphatase indicates the species to be a rather weak competitor for organic phosphate when substrate concentrations are low. *C*. *peruvianus* follows the general trend of northern Adriatic phytoplankton and induces extracellular APA during stratification of the water column and increased primary production in situ but shows a relative reduction of phospholipids as a second adaptation to low phosphate availability. As a result, the species only very rarely achieves noteworthy contributions and never dominate phytoplankton community. The species is able to take up dissolved phosphate resources better during resting phases with low cell division rates which might be a good adaptation to fast changing nutrient regimes or highly structured planktonic ecosystems. Limited availability of PO_4_ induces morphological adaptations, namely the elongation and thickening of setae as well as the elongation of the pervalvar axis that resemble the transition of *C*. *peruvianus* Brightwell to its variety *C*. *peruvianus* var. *robusta* Cleve. This observation suggests that *C*. *peruvianus* var. *robusta* Cleve should be synonymized with *C*. *peruvianus* Brightwell. Morphological change increases the surface area available for alkaline phosphatase improving the species capacity to exploit dissolved organic phosphates. Morphometric analysis of the species in in situ samples might also be a tool to detect stress by limited availability of PO_4_ in seas and oceans. All results combined depict *C*. *peruvianus* Brightwell as a generalist, opportunistic and non-bloom forming diatom species.

## Supporting information

S1 Fig Circumglobal distribution of *C*. *peruvianus* observations accumulated in the global biodiversity information facility GBIF. (TIF)
