# Supplementary figures and images for: Insights into the life strategy of the common marine diatom Chaetoceros peruvianus Brightwell
Source: PLoS One. 2018 Sep 12;13(9):e0203634. doi: 10.1371/journal.pone.0203634 (PMC6135401; doi:10.1371/journal.pone.0203634)

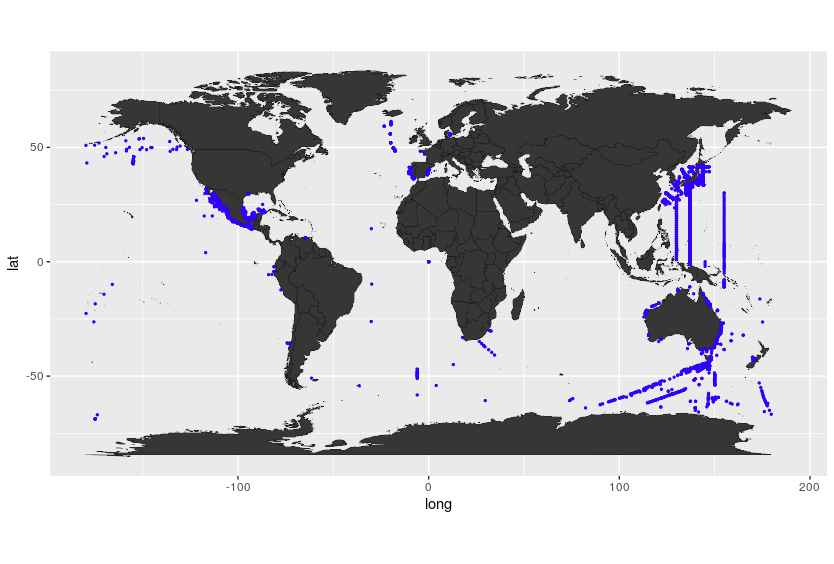

Supplement: S1 Fig — (TIF) [file pone.0203634.s001.tif]
